# Bis[2-(2-pyridylmethyl­amino)ethane­sulfonato-κ^3^ *N*,*N*′,*O*]zinc(II)

**DOI:** 10.1107/S1600536809019990

**Published:** 2009-06-06

**Authors:** Zhong-Xiang Du, Gui-Ying Zhang

**Affiliations:** a Department of Chemistry, Luoyang Normal University, Luoyang, Henan 471022, People’s Republic of China

## Abstract

The title mononuclear complex, [Zn(C_8_H_11_N_2_O_3_S)_2_], is a zinc salt of 2-(2-pyridylmethyl­amino)ethane­sulfonic acid (Hpmt). The Zn^II^ ion is located on an inversion centre and is octahedrally surrounded by four N and two O atoms. The deprotonated pmt^−^ anion coordinates in a facial arrangement through its two N atoms and one of the sulfonate O atoms. The crystal packing is determined by inter­molecular N—H⋯O and C—H⋯O hydrogen bonds.

## Related literature

For the structures of the Co(II) and Ni(II) analogues, see: Li *et al.* (2008 ▶); Liao *et al.* (2007 ▶). For the preparation of the Hpmt ligand, see: Li *et al.* (2006 ▶).
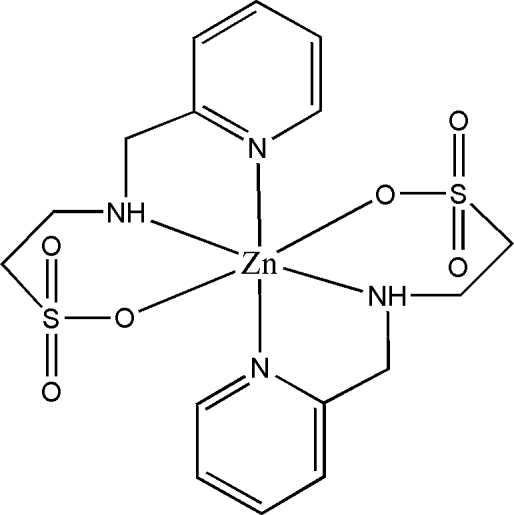


## Experimental

### 

#### Crystal data

[Zn(C_8_H_11_N_2_O_3_S)_2_]
*M* *_r_* = 495.87
Monoclinic, 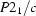

*a* = 9.6288 (13) Å
*b* = 10.0047 (13) Å
*c* = 11.3624 (15) Å
β = 105.965 (1)°
*V* = 1052.4 (2) Å^3^
*Z* = 2
Mo *K*α radiation
μ = 1.41 mm^−1^
*T* = 291 K
0.50 × 0.39 × 0.29 mm

#### Data collection

Bruker APEXII CCD area-detector diffractometer
Absorption correction: multi-scan (*SADABS*; Sheldrick, 1996 ▶) *T*_min_ = 0.540, *T*_max_ = 0.689
6318 measured reflections
2419 independent reflections
2221 reflections with *I* > 2σ(*I*)
*R*_int_ = 0.012

#### Refinement

*R*[*F* ^2^ > 2σ(*F* ^2^)] = 0.023
*wR*(*F* ^2^) = 0.062
*S* = 1.07
2419 reflections
137 parameters
H atoms treated by a mixture of independent and constrained refinement
Δρ_max_ = 0.23 e Å^−3^
Δρ_min_ = −0.38 e Å^−3^

### 

Data collection: *APEX2* (Bruker, 2004 ▶); cell refinement: *SAINT* (Bruker, 2004 ▶); data reduction: *SAINT*; program(s) used to solve structure: *SHELXS97* (Sheldrick, 2008 ▶); program(s) used to refine structure: *SHELXL97* (Sheldrick, 2008 ▶); molecular graphics: *SHELXTL* (Sheldrick, 2008 ▶); software used to prepare material for publication: *SHELXTL*.

## Supplementary Material

Crystal structure: contains datablocks global, I. DOI: 10.1107/S1600536809019990/at2794sup1.cif

Structure factors: contains datablocks I. DOI: 10.1107/S1600536809019990/at2794Isup2.hkl

Additional supplementary materials:  crystallographic information; 3D view; checkCIF report

## Figures and Tables

**Table:** 

Zn1—N2	2.1336 (12)
Zn1—O1	2.1465 (11)
Zn1—N1	2.2130 (13)

**Table:** 

N2—Zn1—O1	92.40 (5)
N2^i^—Zn1—O1	87.60 (4)
N2—Zn1—N1^i^	101.93 (5)
O1—Zn1—N1^i^	89.78 (5)
N2—Zn1—N1	78.06 (5)
O1—Zn1—N1	90.22 (5)

Symmetry code: (i) 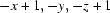.

**Table 2:** Hydrogen-bond geometry (Å, °)

*D*—H⋯*A*	*D*—H	H⋯*A*	*D*⋯*A*	*D*—H⋯*A*
N2—H1*N*⋯O2^ii^	0.855 (18)	2.079 (18)	2.9259 (17)	170.6 (16)
C1—H1⋯O2^iii^	0.93	2.47	3.388 (2)	169
C4—H4⋯O3^iv^	0.93	2.49	3.324 (2)	150
C6—H6*B*⋯O1^i^	0.97	2.56	3.056 (2)	112
C8—H8*B*⋯O2^v^	0.97	2.56	3.265 (2)	130

Symmetry codes: (i) 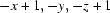; (ii) 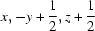; (iii) 
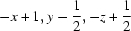
; (iv) 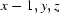; (v) 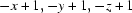.

**Fig. 1.**
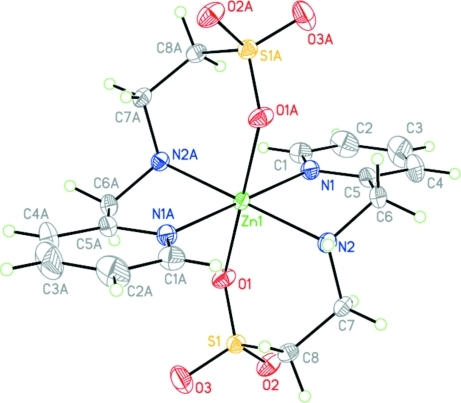
Molecular structure of (I), with displacement ellipsoids drawn at the 30% probability level. Atoms with the suffix A are at the symmetry position (-x, -y, -z).

**Fig. 2.**
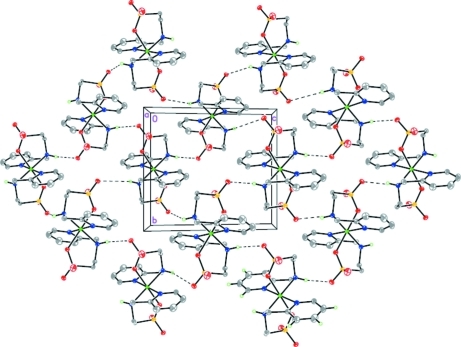
The hydrogen bonding interactions in (I) (dashed lines) projected in bc plane. H atoms on C atoms have been omitted.

## supplementary crystallographic information

### Comment

The complex (I) is isostructural with its analogues [Co(C_8_H_11_N_2_O_3_S)_2_] (Li *et al.*, 2006), [Ni(C_8_H_11_N_2_O_3_S)_2_] (Liao *et al.*, 2007) and [Cu(C_8_H_11_N_2_O_3_S)_2_].2H_2_O (Li *et al.*, 2008), whose structures have been described in detail. The six-coordinate Zn^II^ ion locates on a centre of symmetry with two deprotonated pmt^-^ anions coordinate in a tridentate facial arrangement with its three donor atoms (Fig.1). The bond lengths and angles of (I) are in good agreement with its Co(II) and Ni(II) analogues (Table 1).

The N—H donor and S═O acceptor groups of the pmt ions are involved in hydrogen bonding interactions and forms a two-dimensional network in the *bc* plane (Table 2 and Fig. 2).

### Experimental

The ligand Hpmt was prepared according to the method of Li *et al.*, 2006. To the solution of Hpmt (2.0 mmol, 0.43 g) in water (25 ml), solid ZnCl_2_ (1 mmol, 0.14 g) was added. The resulting mixture was stirred at 333 K for 5 h, then cooled to room temperature. After filtration, the filtrate was left to stand at room temperature for slow evaporation. Colourless block-shaped crystals suitable for X-ray diffraction were obtained in a yield of 42%. Analysis, found: C 38.66, H 4.37, N 11.32, S 12.95%; C_16_H_22_N_4_O_6_S_2_Zn requires: C 38.72, H 4.44, N 11.29, S 12.90%. IR (KBr, ν, cm^-1^): 771.3 [γ(C=C—H)], 746.5(γCH_2_); 1190.3, 1151.4, 1038.8(ν SO_3_^-^); 1607.2, 1572.3(ν C=C + ν C=N); 3198.2(ν N—H).

### Refinement

H atoms bonded to C were positioned geometrically with C—H distance of 0.93–0.97 Å, and treated as riding atoms, with *U*_iso_(H) =1.2*U*_eq_(C). The N—H hydrogen atom was located in a difference Fourier map and refined isotropically.

### Crystal data

**Table:**

[Zn(C_8_H_11_N_2_O_3_S)_2_]	*F*(000) = 512
*M**_r_* = 495.87	*D*_x_ = 1.565 Mg m^−^^3^
Monoclinic, *P*2_1_/*c*	Mo *K*α radiation, λ = 0.71073 Å
Hall symbol: -P 2ybc	Cell parameters from 4168 reflections
*a* = 9.6288 (13) Å	θ = 2.8–28.2°
*b* = 10.0047 (13) Å	µ = 1.41 mm^−^^1^
*c* = 11.3624 (15) Å	*T* = 291 K
β = 105.965 (1)°	Block, colourless
*V* = 1052.4 (2) Å^3^	0.50 × 0.39 × 0.29 mm
*Z* = 2	

### Data collection

**Table:**

Bruker APEXII CCD area-detector diffractometer	2419 independent reflections
Radiation source: fine-focus sealed tube	2221 reflections with *I* > 2σ(*I*)
graphite	*R*_int_ = 0.012
φ and ω scans	θ_max_ = 27.5°, θ_min_ = 2.8°
Absorption correction: multi-scan (*SADABS*; Sheldrick, 1996)	*h* = −11→12
*T*_min_ = 0.540, *T*_max_ = 0.689	*k* = −9→12
6318 measured reflections	*l* = −14→14

### Refinement

**Table:**

Refinement on *F*^2^	Primary atom site location: structure-invariant direct methods
Least-squares matrix: full	Secondary atom site location: difference Fourier map
*R*[*F*^2^ > 2σ(*F*^2^)] = 0.023	Hydrogen site location: inferred from neighbouring sites
*wR*(*F*^2^) = 0.062	H atoms treated by a mixture of independent and constrained refinement
*S* = 1.07	*w* = 1/[σ^2^(*F*_o_^2^) + (0.0322*P*)^2^ + 0.3355*P*] where *P* = (*F*_o_^2^ + 2*F*_c_^2^)/3
2419 reflections	(Δ/σ)_max_ = 0.001
137 parameters	Δρ_max_ = 0.23 e Å^−^^3^
0 restraints	Δρ_min_ = −0.38 e Å^−^^3^

### Special details

**Table:**

Geometry. All e.s.d.'s (except the e.s.d. in the dihedral angle between two l.s. planes)are estimated using the full covariance matrix. The cell e.s.d.'s are takeninto account individually in the estimation of e.s.d.'s in distances, anglesand torsion angles; correlations between e.s.d.'s in cell parameters are onlyused when they are defined by crystal symmetry. An approximate (isotropic)treatment of cell e.s.d.'s is used for estimating e.s.d.'s involving l.s. planes.
Refinement. Refinement of *F*^2^ against ALL reflections. The weighted *R*-factor *wR* and goodness of fit *S* are based on *F*^2^, conventional *R*-factors *R* are based on *F*, with *F* set to zero for negative *F*^2^. The threshold expression of *F*^2^ > σ(*F*^2^) is used only for calculating *R*-factors(gt) *etc*. and is not relevant to the choice of reflections for refinement. *R*-factors based on *F*^2^ are statistically about twice as large as those based on *F*, and *R*- factors based on ALL data will be even larger.

### Fractional atomic coordinates and isotropic or equivalent isotropic displacement parameters (Å^2^)

**Table:**

	*x*	*y*	*z*	*U*_iso_*/*U*_eq_	
Zn1	0.5000	0.0000	0.5000	0.02437 (8)	
S1	0.63235 (4)	0.29294 (4)	0.45567 (3)	0.02858 (10)	
O1	0.57188 (13)	0.16226 (11)	0.40792 (10)	0.0375 (3)	
O2	0.55332 (14)	0.40018 (12)	0.37950 (10)	0.0428 (3)	
O3	0.78676 (14)	0.30037 (16)	0.47547 (13)	0.0573 (4)	
N1	0.27433 (14)	0.03618 (13)	0.39160 (12)	0.0309 (3)	
N2	0.42452 (13)	0.13247 (12)	0.61609 (11)	0.0274 (3)	
C1	0.2143 (2)	0.02195 (18)	0.27020 (16)	0.0412 (4)	
H1	0.2677	−0.0185	0.2232	0.049*	
C2	0.0760 (2)	0.0655 (3)	0.2137 (2)	0.0633 (6)	
H2	0.0370	0.0549	0.1297	0.076*	
C3	−0.0031 (2)	0.1249 (3)	0.2834 (2)	0.0746 (7)	
H3	−0.0961	0.1558	0.2468	0.090*	
C4	0.05670 (19)	0.1385 (2)	0.4085 (2)	0.0584 (5)	
H4	0.0044	0.1775	0.4571	0.070*	
C5	0.19633 (16)	0.09248 (16)	0.45981 (15)	0.0350 (3)	
C6	0.26892 (17)	0.10136 (17)	0.59524 (15)	0.0360 (3)	
H6A	0.2233	0.1707	0.6313	0.043*	
H6B	0.2584	0.0171	0.6342	0.043*	
C7	0.44409 (17)	0.27835 (15)	0.59923 (13)	0.0303 (3)	
H7A	0.4184	0.3273	0.6639	0.036*	
H7B	0.3796	0.3066	0.5217	0.036*	
C8	0.59850 (16)	0.31189 (15)	0.60131 (13)	0.0292 (3)	
H8A	0.6637	0.2542	0.6599	0.035*	
H8B	0.6190	0.4034	0.6287	0.035*	
H1N	0.4634 (18)	0.1133 (17)	0.6913 (16)	0.032 (4)*	

### Atomic displacement parameters (Å^2^)

**Table:**

	*U*^11^	*U*^22^	*U*^33^	*U*^12^	*U*^13^	*U*^23^
Zn1	0.02484 (13)	0.02218 (13)	0.02641 (12)	0.00349 (8)	0.00760 (9)	−0.00102 (8)
S1	0.03239 (19)	0.02599 (19)	0.02848 (17)	0.00050 (14)	0.01027 (14)	0.00143 (13)
O1	0.0591 (7)	0.0267 (6)	0.0308 (5)	−0.0037 (5)	0.0194 (5)	−0.0023 (4)
O2	0.0672 (8)	0.0301 (6)	0.0319 (5)	0.0094 (5)	0.0148 (5)	0.0068 (5)
O3	0.0353 (7)	0.0786 (11)	0.0625 (8)	−0.0049 (6)	0.0208 (6)	−0.0068 (7)
N1	0.0272 (6)	0.0305 (6)	0.0330 (6)	0.0024 (5)	0.0051 (5)	0.0010 (5)
N2	0.0307 (6)	0.0268 (6)	0.0253 (6)	0.0022 (5)	0.0087 (5)	0.0003 (5)
C1	0.0404 (9)	0.0424 (10)	0.0366 (8)	−0.0027 (7)	0.0035 (7)	0.0020 (7)
C2	0.0453 (11)	0.0799 (16)	0.0501 (11)	−0.0037 (11)	−0.0114 (9)	0.0067 (11)
C3	0.0310 (10)	0.097 (2)	0.0817 (16)	0.0137 (11)	−0.0080 (10)	0.0102 (15)
C4	0.0270 (8)	0.0691 (14)	0.0786 (14)	0.0112 (9)	0.0137 (9)	0.0001 (11)
C5	0.0259 (7)	0.0329 (8)	0.0474 (9)	0.0013 (6)	0.0118 (6)	0.0005 (7)
C6	0.0327 (8)	0.0387 (9)	0.0430 (8)	0.0013 (7)	0.0210 (7)	−0.0030 (7)
C7	0.0367 (8)	0.0245 (7)	0.0313 (7)	0.0042 (6)	0.0120 (6)	−0.0028 (6)
C8	0.0348 (7)	0.0268 (7)	0.0241 (6)	−0.0009 (6)	0.0048 (5)	−0.0029 (5)

### Geometric parameters (Å, °)

**Table:**

Zn1—N2	2.1336 (12)	C1—H1	0.9300
Zn1—N2^i^	2.1336 (12)	C2—C3	1.376 (4)
Zn1—O1	2.1465 (11)	C2—H2	0.9300
Zn1—O1^i^	2.1465 (11)	C3—C4	1.386 (3)
Zn1—N1^i^	2.2130 (13)	C3—H3	0.9300
Zn1—N1	2.2130 (13)	C4—C5	1.388 (2)
S1—O3	1.4431 (13)	C4—H4	0.9300
S1—O2	1.4549 (12)	C5—C6	1.508 (2)
S1—O1	1.4727 (11)	C6—H6A	0.9700
S1—C8	1.7825 (15)	C6—H6B	0.9700
N1—C5	1.342 (2)	C7—C8	1.518 (2)
N1—C1	1.349 (2)	C7—H7A	0.9700
N2—C6	1.4841 (19)	C7—H7B	0.9700
N2—C7	1.4908 (19)	C8—H8A	0.9700
N2—H1N	0.856 (17)	C8—H8B	0.9700
C1—C2	1.380 (3)		
			
N2—Zn1—N2^i^	180.0	N1—C1—H1	119.0
N2—Zn1—O1	92.40 (5)	C2—C1—H1	119.0
N2^i^—Zn1—O1	87.60 (4)	C3—C2—C1	118.96 (19)
N2—Zn1—O1^i^	87.60 (5)	C3—C2—H2	120.5
N2^i^—Zn1—O1^i^	92.40 (5)	C1—C2—H2	120.5
O1—Zn1—O1^i^	180.0	C2—C3—C4	119.55 (18)
N2—Zn1—N1^i^	101.93 (5)	C2—C3—H3	120.2
N2^i^—Zn1—N1^i^	78.06 (5)	C4—C3—H3	120.2
O1—Zn1—N1^i^	89.78 (5)	C3—C4—C5	118.7 (2)
O1^i^—Zn1—N1^i^	90.22 (5)	C3—C4—H4	120.7
N2—Zn1—N1	78.06 (5)	C5—C4—H4	120.7
N2^i^—Zn1—N1	101.94 (5)	N1—C5—C4	121.82 (16)
O1—Zn1—N1	90.22 (5)	N1—C5—C6	115.98 (13)
O1^i^—Zn1—N1	89.78 (5)	C4—C5—C6	122.19 (16)
N1^i^—Zn1—N1	180.0	N2—C6—C5	109.85 (12)
O3—S1—O2	113.74 (9)	N2—C6—H6A	109.7
O3—S1—O1	112.90 (8)	C5—C6—H6A	109.7
O2—S1—O1	110.27 (7)	N2—C6—H6B	109.7
O3—S1—C8	107.06 (8)	C5—C6—H6B	109.7
O2—S1—C8	105.93 (7)	H6A—C6—H6B	108.2
O1—S1—C8	106.36 (7)	N2—C7—C8	111.86 (12)
S1—O1—Zn1	129.76 (6)	N2—C7—H7A	109.2
C5—N1—C1	118.96 (14)	C8—C7—H7A	109.2
C5—N1—Zn1	111.53 (10)	N2—C7—H7B	109.2
C1—N1—Zn1	129.14 (12)	C8—C7—H7B	109.2
C6—N2—C7	110.03 (12)	H7A—C7—H7B	107.9
C6—N2—Zn1	105.77 (9)	C7—C8—S1	112.98 (10)
C7—N2—Zn1	116.88 (9)	C7—C8—H8A	109.0
C6—N2—H1N	104.9 (12)	S1—C8—H8A	109.0
C7—N2—H1N	108.2 (12)	C7—C8—H8B	109.0
Zn1—N2—H1N	110.4 (11)	S1—C8—H8B	109.0
N1—C1—C2	122.03 (19)	H8A—C8—H8B	107.8
			
O3—S1—O1—Zn1	103.94 (10)	N1—Zn1—N2—C7	−90.09 (10)
O2—S1—O1—Zn1	−127.61 (9)	C5—N1—C1—C2	1.1 (3)
C8—S1—O1—Zn1	−13.19 (11)	Zn1—N1—C1—C2	−171.19 (16)
N2—Zn1—O1—S1	33.74 (10)	N1—C1—C2—C3	−0.2 (3)
N2^i^—Zn1—O1—S1	−146.26 (10)	C1—C2—C3—C4	−0.7 (4)
N1^i^—Zn1—O1—S1	−68.19 (10)	C2—C3—C4—C5	0.7 (4)
N1—Zn1—O1—S1	111.81 (10)	C1—N1—C5—C4	−1.1 (3)
N2—Zn1—N1—C5	−14.41 (11)	Zn1—N1—C5—C4	172.45 (15)
N2^i^—Zn1—N1—C5	165.59 (11)	C1—N1—C5—C6	178.25 (15)
O1—Zn1—N1—C5	−106.82 (11)	Zn1—N1—C5—C6	−8.16 (17)
O1^i^—Zn1—N1—C5	73.18 (11)	C3—C4—C5—N1	0.3 (3)
N2—Zn1—N1—C1	158.35 (15)	C3—C4—C5—C6	−179.1 (2)
N2^i^—Zn1—N1—C1	−21.65 (15)	C7—N2—C6—C5	80.21 (15)
O1—Zn1—N1—C1	65.94 (14)	Zn1—N2—C6—C5	−46.87 (14)
O1^i^—Zn1—N1—C1	−114.06 (14)	N1—C5—C6—N2	37.7 (2)
O1—Zn1—N2—C6	122.46 (10)	C4—C5—C6—N2	−142.93 (17)
O1^i^—Zn1—N2—C6	−57.54 (10)	C6—N2—C7—C8	−171.67 (11)
N1^i^—Zn1—N2—C6	−147.26 (9)	Zn1—N2—C7—C8	−51.08 (14)
N1—Zn1—N2—C6	32.74 (9)	N2—C7—C8—S1	85.42 (13)
O1—Zn1—N2—C7	−0.37 (10)	O3—S1—C8—C7	−168.32 (11)
O1^i^—Zn1—N2—C7	179.63 (10)	O2—S1—C8—C7	69.97 (12)
N1^i^—Zn1—N2—C7	89.91 (10)	O1—S1—C8—C7	−47.37 (12)

Symmetry codes: (i) −*x*+1, −*y*, −*z*+1.

### Hydrogen-bond geometry (Å, °)

**Table:**

*D*—H···*A*	*D*—H	H···*A*	*D*···*A*	*D*—H···*A*
N2—H1N···O2^ii^	0.855 (18)	2.079 (18)	2.9259 (17)	170.6 (16)
C1—H1···O2^iii^	0.93	2.47	3.388 (2)	169
C4—H4···O3^iv^	0.93	2.49	3.324 (2)	150
C6—H6B···O1^i^	0.97	2.56	3.056 (2)	112
C8—H8B···O2^v^	0.97	2.56	3.265 (2)	130

Symmetry codes: (ii) *x*, −*y*+1/2, *z*+1/2; (iii) −*x*+1, *y*−1/2, −*z*+1/2; (iv) *x*−1, *y*, *z*; (i) −*x*+1, −*y*, −*z*+1; (v) −*x*+1, −*y*+1, −*z*+1.
